# Primary Transanal Management of Rectal Atresia in a Neonate 

**Published:** 2016-04-10

**Authors:** Braiek M, Ksia A, Krichen I, Belhassen S, Maazoun K, Ben youssef S, Kechiche N, Mekki M, Nouri A

**Affiliations:** Pediatric Surgery Department, Fattouma Bourguiba Hospital, Monastir University, Tunisia

**Keywords:** Rectal atresia, Transanal repair, Neonate

## Abstract

Rectal atresia (RA) with a normal anus is a rare anomaly. We describe a case of rectal atresia in a newborn male presenting with an abdominal distension and failure of passing meconium. The rectal atresia was primarily operated by transanal route.

## CASE REPORT

A 2-day-old boy admitted in our department with abdominal distension, bilious vomiting, and failure to pass meconium. He was born spontaneously at full term after an uncomplicated pregnancy with a birth weight of 3800g. Several prenatal ultrasonic investigations were reported as normal. Clinical examination revealed abdominal distension, with a normal perineum, genitalia and anus identified in the correct location in the perineum. Digital rectal examination with catheter showed a blind ending anal canal (2-3cm long). X-ray (invertogram with Hegar dilator passed through anal opening) showed dilator abutting the air column in the bowel. The neonate was diagnosed with rectal atresia. Echocardiography and abdominal ultrasonography did not reveal any associated anomaly. At operation, the mucosal web (type I rectal atresia) was identified and exposed by transanal approach (Fig. 1): Mucosal web was bulging significantly due to meconium behind it. The mucosal web was excised with cautery which revealed meconium from the upper colonic segment. Few stitches were placed at the site of mucosectormy for hemostasis and to prevent re-adhesion (Fig. 2). The postoperative recovery was uneventful. We began anal dilations at 2 weeks post operatively. After a follow up period of 6 months, the baby is well, with normal bowel functions and no anal stricture. 

**Figure:**
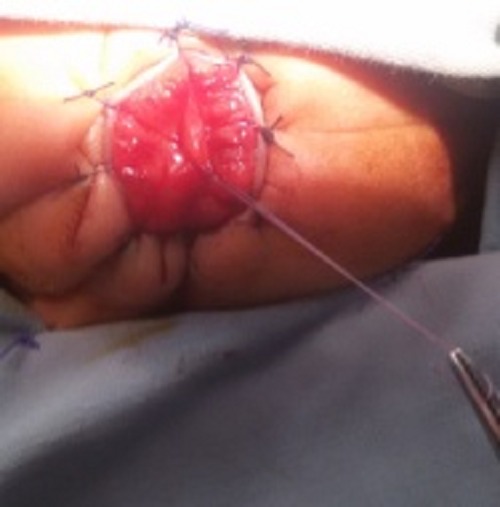
Figure 1: Intra operatively, an inner mucosal membrane was seen bulging.

**Figure:**
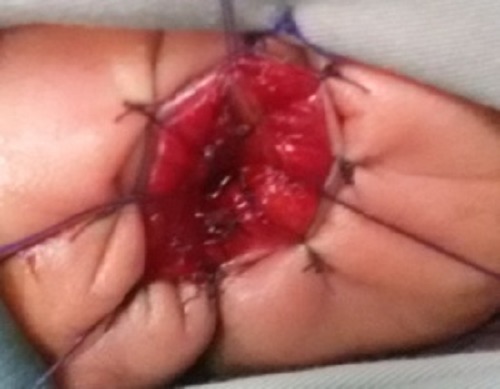
Figure 2: After transanal excision of web

## DISCUSSION

Rectal atresia is a rare anorectal anomaly combining a normally developed anus and an atretic rectal segment representing 1-2% of all anorectal anomalies. Rectal atresia is considered separate from imperforate anus or anal atresia because, in rectal atresia, the anus is present and normal, but a variable rectal segment is atretic [1].

Failure to pass meconium, progressive abdominal distention, refusal to feed and vomiting suggest intestinal obstruction in neonates and lead to further investigations [2]. The clinical diagnosis is easy to confirm. When a size 8 rubber catheter is passed per anus, it stops 2-3cm from anal verge [2]. In our case: red rubber catheter could not be passed beyond 3 cm of anal verge. However, the deceptively normal appearance of the anus and perineum may delay the diagnosis until the massive abdominal distension occurs. Inability to pass a rectal thermometer may be the first sign [3]. 

The rectal atresia can be managed in single stage as well as staged procedures. Various approaches like posterior sagittal approach and rectal end-to-end anastomosis, abdomino-posterior sagittal approach, and transanal approach [3]. In our case, the rectal atresia was type I i.e. having a mucosal web which was managed successfully via transanal approach.

## Footnotes

**Source of Support:** Nil

**Conflict of Interest:** Nil
